# The Transformation of Adaptation Specificity to Whisker Identity from Brainstem to Thalamus

**DOI:** 10.3389/fnsys.2016.00056

**Published:** 2016-06-23

**Authors:** Muna Jubran, Boaz Mohar, Ilan Lampl

**Affiliations:** Department of Neurobiology, Weizmann Institute of ScienceRehovot, Israel

**Keywords:** thalamus, VPM, SSA, adaptation, whisker, *in vivo*, PrV, somatosensory

## Abstract

Stimulus specific adaptation has been studied extensively in different modalities. High specificity implies that deviant stimulus induces a stronger response compared to a common stimulus. The thalamus gates sensory information to the cortex, therefore, the specificity of adaptation in the thalamus must have a great impact on cortical processing of sensory inputs. We studied the specificity of adaptation to whisker identity in the ventral posteromedial nucleus of the thalamus (VPM) in rats using extracellular and intracellular recordings. We found that subsequent to repetitive stimulation that induced strong adaptation, the response to stimulation of the same, or any other responsive whisker was equally adapted, indicating that thalamic adaptation is non-specific. In contrast, adaptation of single units in the upstream brainstem principal trigeminal nucleus (PrV) was significantly more specific. Depolarization of intracellularly recorded VPM cells demonstrated that adaptation is not due to buildup of inhibition. In addition, adaptation increased the probability of observing complete synaptic failures to tactile stimulation. In accordance with short-term synaptic depression models, the evoked synaptic potentials in response to whisker stimulation, subsequent to a response failure, were facilitated. In summary, we show that local short-term synaptic plasticity is involved in the transformation of adaptation in the trigemino-thalamic synapse and that the low specificity of adaptation in the VPM emerges locally rather than cascades from earlier stages. Taken together we suggest that during sustained stimulation, local thalamic mechanisms equally suppress inputs arriving from different whiskers before being gated to the cortex.

## Introduction

Adaptation in primary sensory cortical areas is usually stimulus specific (Marlin et al., 1991; Ulanovsky et al., 2003; Katz et al., 2006), i.e., rare or deviant stimuli induce stronger responses compared to common stimuli. However, much less is known about the specificity of adaptation in the thalamus. Some studies suggest that adaptation in the auditory thalamus is highly specific (Anderson et al., 2009; Antunes et al., 2010), whereas in the visual thalamus, adaptation is much less specific both to spatial frequency (Duong and Freeman, 2007) and to orientation (Dhruv and Carandini, 2014).

It was proposed that the main role of the thalamus is to gate or modulate ascending information to the cortex (Steriade et al., 1993; McCormick and Bal, 1994; Sherman and Guillery, 1996; Aguilar and Castro-Alamancos, 2005). In the somatosensory system, tactile information ascends from the brainstem principal trigeminal nucleus (PrV) to the ventral posteromedial nucleus of the thalamus (VPM) as part of the lemniscal pathway that innervates layer 4 of the barrel cortex (Diamond et al., 2008). Most VPM neurons have receptive fields (RFs) comprised of one principal (PW) and several adjacent whiskers (AWs; Simons and Carvell, 1989; Friedberg et al., 1999; Katz et al., 2012). VPM neurons are mainly driven by direct glutamatergic monosynaptic inputs from homologous “barrelette” cells located in the PrV (Magnusson et al., 1987; Castro-Alamancos, 2002a). Lesion studies suggest that multi-whisker RF synthesis might occur in the PrV through intersubnuclear projections from the interpolar nucleus of the trigeminal complex (SpVi; Rhoades et al., 1987; Friedberg et al., 1999; Timofeeva et al., 2004). Moreover, inspection of PrV projections revealed that there are as few as 1–3 trigemino-thalamic axons converging on a single VPM cell (Castro-Alamancos, 2002a; Deschênes et al., 2003). Thus, thalamic multi-whisker RFs could be directly inherited from PrV multi-whisker cells via trigemino-thalamic fibers. However, they could also result from the convergence of single-whisker PrV axons on VPM cells. Hence, the innervation pattern of VPM cells by PrV inputs could play an important role in determining the specificity of adaptation in the VPM to whisker identity.

We investigated the specificity of adaptation to whisker identity in VPM neurons at suprathreshold and subthreshold levels. Since rats can move neighboring whiskers independently (Sachdev et al., 2002; Knutsen et al., 2006), the specificity of adaptation to whisker identity might be highly relevant to behavior. We found that firing response to test stimulation of any whisker was equally suppressed by prior adapting stimulation of the same, or any other responsive whisker. Non-specific adaptation was also evident in the subthreshold feedforward synaptic inputs. Recordings from upstream PrV neurons showed that adaptation in the brainstem is more specific compared to the VPM, in particular for AW stimulation. Hence, local thalamic mechanisms contribute to the non-specificity of VPM adaptation. Failure analysis of synaptic responses during adaptation strongly supports the idea that short-term synaptic depression at the trigemino-thalamic synapse contributes to VPM adaptation. Our results argue that the specificity of adaptation to whisker identity in the thalamus is not inherited from earlier stages and that synaptic depression contributes to thalamic adaptation.

## Materials and Methods

### Animal Preparation

All surgical and experimental procedures were conducted in accordance with the regulations of the Weizmann Institute Animal Care and Use Committee. Female adult Wistar rats (100 ± 20 g, *n* = 21) were initially anesthetized with ketamine (100 mg/kg) and xylasine (20 mg/kg) mixture injected intraperitoneally and a tracheotomy was performed. Rats were then mounted in a stereotaxic device and respirated with oxygen-enriched air. The levels of end-tidal CO_2_ and heart rate were monitored throughout the experiment and body temperature was maintained at 36.5 ± 0.5°C using a heating blanket and a rectal thermometer. The depth of anesthesia was monitored by assessing EEG signals, heart rate, corneal and pinch withdrawal reflexes and was maintained at stage III/3–4 (Friedberg et al., 1999) by supplementary intraperitoneal injections of ketamine (50% in saline).

### Electrophysiological Recordings

Extracellualr and intracellular recordings from the VPM and unit recordings from the PrV were performed as previously reported (Mohar et al., 2013, 2015).

#### Thalamic Recordings

For thalamic recordings, the skull was exposed and a craniotomy (0.5–1 mm in diameter) was made above the VPM of the thalamus (3 mm posterior and 2.7–3.2 mm lateral to bregma) and a portion of the dura mater was carefully removed. Extracellular single-unit recordings were performed with high resistance (30–40 MΩ) borosilicate micropipettes (o.d., 1.5 mm; i.d., 0.86 mm; Sutter Instruments, Novato, CA, USA), pulled with a P-97 micropipette puller (Sutter Instruments) and filled with 2 M K-acetate. Intracellular “Blind” sharp recordings were obtained using high resistance (60–100 MΩ) borosilicate micropipettes (o.d., 1.5 mm; i.d., 0.86 mm; Sutter Instruments, Novato, CA, USA), pulled with a P-97 micropipette puller (Sutter Instruments) and filled with 2 M K-acetate. To assess the synaptic inputs of VPM cells QX-314 (30–50 mM) was added to the intracellular solution to prevent action potentials. Micropipettes were vertically advanced to the VPM and recordings were made at a depth of 4.3–5.2 mm. To protect the brain from drying, we covered the craniotomy with a few drops of artificial cerebrospinal fluid (ACSF). Signals were amplified using Axoclamp-2B (Axon Instruments, Foster City, CA, USA) and digitized at 20 kHz.

#### Brainstem PrV Extracellular Unit Recordings

The skin was removed from the top and rostral parts of the head and the dorsal part of the cerebellum and the first cervical segment of the spinal cord were exposed. The dura was removed and the area was continuously washed with ACSF. Extracellular recordings from PrV cells were obtained using 1 MΩ tungsten microelectrodes (AM-573220; A-M Systems, Sequim, WA, USA). Electrodes were inserted into the exposed brainstem at an angle of 2–7° in relation to the horizontal plane and in parallel to the sagittal plane, starting 2–3 mm lateral to the midline and 1 mm dorsal to the meeting point of the brainstem and cerebellum. PrV units were detected after advancing the electrode 3 mm from the insertion point (Mohar et al., 2013, 2015). Extracellular signals were amplified using a model 3000 amplifier (A-M Systems, Sequim, WA, USA), bandpassed at 0.3–3 kHz and amplified between 1000–10,000 before digitized at 10 kHz using homemade acquisition software in LabView (National Instruments, Austin, TX, USA). Single units were isolated using WaveClus software (Quiroga et al., 2004) in Matlab (Mathworks, Natick, MA, USA).

### Whisker Stimulation

Whiskers were mechanically stimulated using a galvanometer servo control motor (6210H; Cambridge Technology Inc., USA) with the matching servo driver and controller (MicroMax 677xx; Cambridge Technology Inc., USA). The displacement near the tip of the metal pipette was measured off-line using an optical displacement measuring system (optoNCDT 1605; Micro-Epsilon). Direction of displacement was always rostrocaudal. Mapping of the RFs was initially done using a hand-held probe after the whiskers were trimmed at a distance of about 10–15 mm from the base. Whisker deflection was obtained by delivering fast-rising voltage commands to evoke fast deflection (~70 mm/s) with a constant rise time of ~1 ms followed by a 10 ms ramp-down signal that returned the whisker to the baseline position. Once a responsive whisker had been identified, all five neighboring whiskers were examined in order to decide if to include the cell in the multi-whisker RF classification analysis. The distinction of the PW was made off-line according to the latency of the evoked excitatory post synaptic potential (EPSP; Brecht and Sakmann, 2002) which was defined, for VPM recordings, as the time measured from whisker stimulation to the point where EPSP reaches 5% of its peak response. Response latency was also used to determine the PW for extracellular VPM and PrV recordings. RFs were mapped for 55 VPM neurons; 31 demonstrated multi-whisker RF whereas 24 neurons responded only to single-whisker stimulation.

### EEG Recordings

To assess the depth of anesthesia during thalamic recordings (Friedberg et al., 1999; Katz et al., 2012), EEG was recorded using low impedance PFA-coated stainless steel wires (diameter 0.003”, A-M systems, WA, USA) which were located in the contralateral parietal cortex.

The fast Fourier transform (FFT) algorithm was used to compute the EEG power spectrum. The depth of anesthesia during the recording of each cell was estimated using an EEG index that was calculated as follows: for each EEG trace recorded simultaneously with VPM data (2.5 s), the power of high alpha frequencies (8–12 Hz) was divided by the power of low delta frequencies (0–4 Hz) and averaged across all repetitions.

### Histology

**Figure 1:**
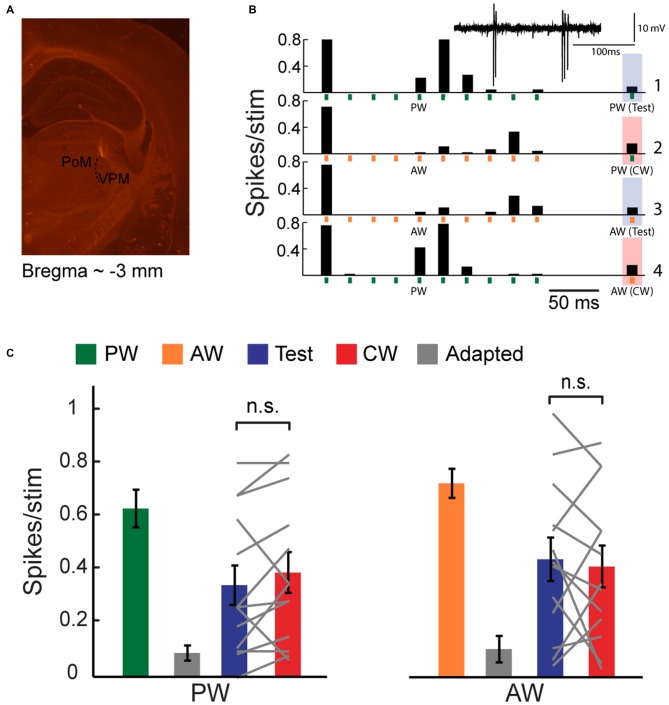
**Cross-whisker (CW) firing adaptation of multi-whisker ventral posteromedial nucleus of the thalamus (VPM) neurons. (A)** Image of a coronal section (30 μm; ~3 mm from Bregma) depicting recording electrode placement. Electrode was coated with DiI and advanced to the VPM. Posteromedial nucleus of the thalamus (PoM). **(B)** Peri-stimulus time histogram (PSTH) of an example VPM neuron (*n* = 40 repetitions) to four different stimulation patterns. Stimuli are depicted below by vertical bars. The response to an isolated principal-whisker (PW) stimulation (B1–2) and adjacent-whisker (AW) stimulation (B3–4) was examined 100 ms following a repetitive stimulation at 40 Hz delivered to the same whisker (test condition, blue shaded area), or to a neighboring whisker (CW condition, red shaded area). Inset: raw single unit recording of the same example cell. **(C)** Population average (*n* = 13 cells) of the number of spikes evoked per stimulus in response to control (*green and orange*), adapted (*gray*, 10th stimulus), test (*blue*) and CW (*red*) stimuli of the PW (*left panel*) and of the AW (*right panel*). Test responses were significantly larger than adapted responses for PW (*p* < 0.02) and AW (*p* < 0.001) stimulation. No significant differences were found between test and CW responses for both whiskers. Each gray line represents the values of a single unit. The error bars represent SEMs.

In a subset of extracellular recording experiments, the tip of the micropipette was coated with DiI (DiIC18(3), life technologies) for placement reconstruction (Figure 1A). At the end of the experiment, animals were deeply anesthetized with pentobarbital sodium and perfused transcardially with PBS followed by 2.5% paraformaldehyde. Brains were post-fixed and cryo-preserved in 2.5% paraformaldehyde and 30% sucrose in PBS, respectively. Subsequently, coronal sections of the brain were obtained (30 μm), mounted on microscope slides and covered with cover glass. Visualization was carried out under a fluorescence microscope system (Leica).

### Data Analysis

The analysis of the recordings was performed using custom software written in Matlab. Amplitude of EPSPs was calculated as the peak voltage response at 4–20 ms after stimulation, minus the mean baseline voltage averaged in 8 ms window centered at the time of stimulation, prior to the onset of the EPSP response. Amplitudes of less than 1 mV above noise level were categorized as failures.

### Statistical Analysis

In all analysis, we did not assume a normal distribution of the data. We therefore used the two sided Wilcoxon signed-rank test. For comparisons in which the number of samples in the two conditions was not equal, we used the two sided Mann-Whitney *U*-test. When more than two groups were compared, Friedman then Wilcoxon signed-rank test for *post hoc* was conducted with a Bonferroni correction applied. Data are presented as mean ± SEM, unless stated otherwise. The critical significance level α was set to 0.05.

## Results

### Adaptation in the VPM is Non-Specific

Extracellular and intracellular membrane potentials were recorded from VPM cells. The location of the recording was verified on-line by the properties of the evoked response to whisker deflections (i.e., short latency and frequency following ability). Also, in a subset of recordings, the tip of the recording micropipette was coated with DiI for placement reconstruction.

We used an oddball paradigm to investigate the specificity of adaptation of multi-whisker VPM cells to tactile stimulation of different whiskers in lightly anesthetized rats. We delivered four different stimulation patterns to two selected whiskers in the RF of recorded VPM neurons (see Figure 1B). The first was a train of 10 brief stimuli at 40 Hz, delivered to one of the responsive whiskers, followed by an isolated stimulation of the same whisker 100 ms later (test condition). The second was similar except the isolated stimulation was now delivered to another whisker (cross-whisker condition; CW). In the two other patterns, we exchanged the identities of the whiskers. The four different stimulation patterns were pseudo-randomly delivered with a 2.5 s interval between trials. We set stimulation frequency for adaptation to 40 Hz in order to get a significant adaptation. The test and CW stimuli were delivered at a longer interval of 100 ms in order to assess the partial recovery from adaptation. We then analyzed the effect of whisker identity; an equal reduction in CW response compared to the test response of the same whisker would indicate that adaptation is not specific.

Extracellular single-unit recordings of an example cell (Figure 1B) show that a repetitive whisker stimulation at 40 Hz results with a substantial firing adaptation of PW (green vertical bars) AW (orange vertical bars). PW and AW were determined by measuring the response latency (5.7 ± 0.5 ms and 7.6 ± 0.3 ms for PW and AW stimulation, *n* = 11, *p* < 0.005, Wilcoxon signed-rank test). Test stimulation of a similar intensity applied to the same whisker (PW or AW) 100 ms after termination of the adapting stimulation revealed only a minor recovery from adaptation (shaded blue bars). Importantly, for both whiskers, the CW response (shaded red bars) was reduced almost to the same level as the test response. The population average of spike count response to the 1st (green and orange bars), 10th (adapted state, gray bars), test (blue bars) and CW (red bars) stimuli are presented in Figure 1C. These data show that the test and CW responses were adapted and were significantly smaller than first response (PW: *p* < 0.02 and *p* < 0.01; AW: *p* < 0.01 and *p* < 0.001 for test and CW responses respectively, *n* = 13, Wilcoxon signed-rank test). Importantly, the test and CW responses were statistically indistinguishable (*P* > 0.05), implying that adaptation adjusts the output spiking response of VPM cells to a constant level regardless of the whisker that was previously stimulated to induce adaptation. This was further analyzed by calculating the adaptation specificity index (ASI; Ulanovsky et al., 2003):

$$ASI\;=\;abs\left(\frac{CW-test}{CW+test}\right)$$

where *CW* and *test* correspond to the firing response for CW and test stimulus, respectively. In this index, a value close to zero implies non-specific adaptation whereas a large positive value indicates that the CW response was higher than the test, resulting with specific adaptation. We found very low ASI values for both PW and AW (0.13 ± 0.1 and 0.015 ± 0.13 for PW and AW, respectively, *n* = 13). A global suppression of evoked responses could result from non-specific inhibition, or intrinsic mechanisms that shift the membrane potential towards a more hyperpolarized state (Sanchez-Vives et al., 2000). However, it is also consistent with the concept that a single VPM cell receives contacts from a small number of multi-whisker PrV cells (Castro-Alamancos, 2002a; Deschênes et al., 2003). Accordingly, adaptation caused by stimulation of one whisker would also lead to synaptic depression of inputs from other responsive whiskers.

**Figure 2:**
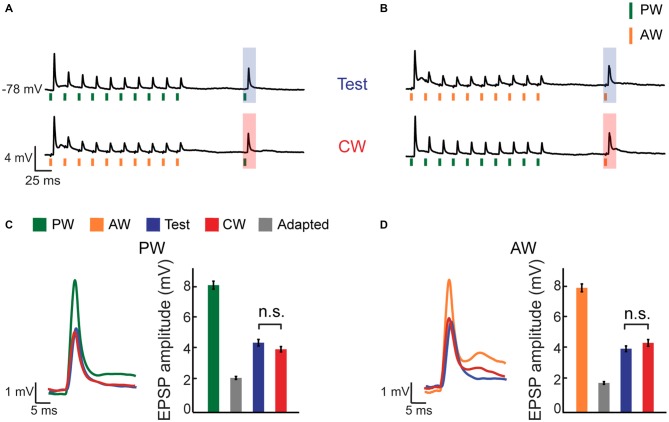
**CW adaptation of an intracellularly recorded multi-whisker VPM neuron. (A,B)** Average membrane potential responses of an example VPM neuron to four different stimulation patterns. Stimuli are depicted below the average traces (*n* = 16 repetitions). **(A)** The response to an isolated PW stimulation was examined 100 ms following repetitive stimulation at 40 Hz (10 stimuli) delivered to the same whisker (test, *blue* shaded area; *upper trace*), or following repetitive stimulation of an AW (CW, *red* shaded area; *lower trace*). **(B)** Same as in **(A)** but for the AW. **(C)** Left panel: overlaid average excitatory post synaptic potentials (EPSPs) to PW stimulation in response to first stimulus in adaptation train, to test and to CW stimulations. Right panel: bar plots of average EPSP amplitudes to first stimulus in adaptation train, adapted (10th stimulus), test and CW stimulations (color coded as in the legend inset in **A**). **(D)** Similar to **(C)** but for the AW.

In order to inspect the contribution of synaptic inputs from the PrV to the global suppression effect, we used sharp intracellular electrodes (+QX-314) and recorded subthreshold membrane potentials of multi-whisker VPM cells (56% of all intracellularly recorded cells) and delivered the same stimulation protocol. PW and AW were determined by measuring the EPSP latency (6.2 ± 0.5 ms and 8.5 ± 0.6 ms for PW and AW stimulation, *n* = 17, *p* < 0.0005, Wilcoxon signed-rank test, see “Materials and Methods” Section), with agreement with previous studies which demonstrated that responses of neurons in the PrV to stimulation of AWs are somewhat delayed relatively to the PW (Brecht and Sakmann, 2002; Minnery et al., 2003; Timofeeva et al., 2004). Unless stated otherwise, cells were recorded without current injection (mean resting potential −66.2 ± 2.3 mV, *n* = 17). The average membrane potential response of an example cell (Figures 2A–D) shows that repetitive stimulation of the PW (green vertical bars) or the AW (orange vertical bars) induced profound adaptation of EPSPs. Significant reduction (i.e., only partial recovery) was found both for the test and CW responses, which are marked by blue and red shaded areas respectively (Figures 2A,B). Importantly, despite the profound reduction in amplitude of the EPSPs in this cell compared to the non-adapted responses (green and orange bars in Figures 2C,D for PW and AW stimulation, respectively), the amplitude of the test and CW EPSPs were statistically indistinguishable both for the PW and AW (blue and red bars in Figures 2C,D), although both significantly recovered from the adapted state (10th stimulus, gray bars in Figures 2C,D, *n* = 16, *p* < 0.001, Wilcoxon signed-rank test). The average EPSPs are depicted together in Figures 2C,D (*left panels*) showing that test and CW response have similar shapes. Thus, for this cell the EPSP response to stimulation of a whisker was equally suppressed by repetitive stimulation of the same or the AW. This indicates that similar to spike count, adaptation equalized the synaptic response, regardless of the whisker that was recently stimulated.

**Figure 3:**
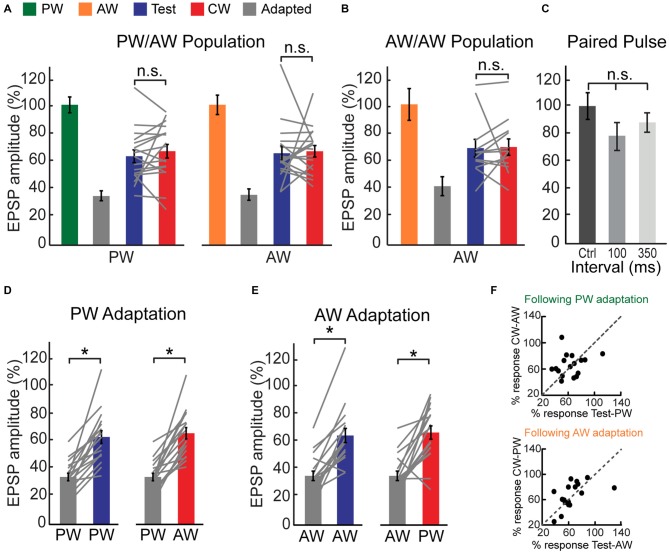
**CW adaptation of intracellularly recorded multi-whisker VPM neurons. (A)** Population average of the EPSP amplitude ratio of 17 multi-whisker neurons in response to adapted, test and CW stimuli of the PW (*left panel*) and of the AW (*right panel*). Test responses were significantly larger than adapted responses for PW (*p* < 0.001) and AW (*p* < 0.001) stimulation. No significant differences were found between test and CW responses for both whiskers. Values are normalized to the first response in adaptation train for both PW and AW (100%, *green/orange*). **(B)** As in **(A)** but for five multi-whisker neurons in response to test, adapted and CW stimuli of an AW (following adaptation of another AW). Test response was significantly larger than adapted response (*p* < 0.05). No significant difference was found between test and CW responses. Each gray line represents the values of a single comparison. **(C)** Paired pulse stimulation of two isolated stimuli delivered to different whiskers reveals no significant interactions as revealed by average amplitude ratio of EPSPs in response to the second deflection with inter-stimulus intervals of 100 (*n* = 6) and 350 ms (*n* = 3). Values are normalized to the amplitude of the response for a given whisker when it was stimulated first. **(D)** Population average of the EPSP amplitude ratio in response to test stimuli of the PW (*left panel*) and CW stimuli of the AW (*right panel*) following adaptation of the PW (*gray bars*). **(E)** Population average of the EPSP amplitude ratio in response to test stimuli of the AW (*left panel*) and CW stimuli of the PW (*right panel*) following adaptation of the AW (*gray bars*). **(F)** Adaptation pressure plots, comparing the amount of suppression made by repetitively stimulating a given whisker on the response to subsequent stimulation of the same, or other whisker. Upper panel—relative EPSP amplitude evoked by stimulation of the PW (test) and AW (CW) following repetitive stimulation of the PW. Lower panel—similar comparison following repetitive stimulation of the AW. Asterisks (*)indicate significant differences (*p* < 0.05). The error bars represent SEMs.

This non-specific suppressive effect of adaptation is also observed at the population level (Figure 3A). While the amplitude of test and CW evoked responses were significantly smaller compared to control response, they were statistically indistinguishable (blue vs. red bars, *n* = 17 cells, *p* > 0.05). This similarity is not due to averaging across the population, as no significant differences in the amplitudes of the test and CW responses were found in the majority of within-cell comparisons (12/17 and 14/17 for the PW and AW respectively, *p* > 0.05). Insignificant differences between the test and the CW responses were also found for interactions between different AWs, not involving the PW. For these recordings, adaptation induced by any AW repetitive stimulation significantly reduced the response to the deflection of the same, or other AW, in a reciprocal manner (Figure 3B, *p* > 0.05, *n* = 12 comparisons in five cells, Wilcoxon signed-rank test). As expected, we found very low ASI values for both PW and AW (0.020 ± 0.03 and 0.015 ± 0.04 for PW and AW, respectively). Furthermore, ASI’s for PW and AW were not correlated in the population (*r* = −0.32, *p* = 0.2).

Similar to the reported results for 100 ms time interval, *CW* and *test* responses were not statistically different when delivered at a shorter interval of 25 ms—the same interval as of the adaptation train (i.e., 25 ms, 1/40 Hz; “Supplementary Figure 1”, *n* = 4, *p* > 0.05).

Next we examined whether the partial recovery from adaptation after 100 ms can be also explained by interactions between the last stimulus of the adaptation train and the test or CW stimulus, as expected for deflection of two whiskers at shorter intervals (Simons and Carvell, 1989). However, in the absence of adaptation, we found no significant reduction in the second response for PW following a single AW stimulation, or* vice versa* (Figure 3C). Altogether, our results strongly suggest that tactile inputs from different whiskers are gated in the VPM by a common adaptation mechanism.

Adaptation of the PW has equivalent effects on PW or AW recovery (i.e., compare the blue bar in the left panel to the red bar of the right panel in Figure 3A). Similarly, the AW test and PW CW responses following repetitive stimulation of the AW are comparable. A cell by cell analysis (Figures 3D–F) shows that the adaptation “pressure”, exerted by repetitive stimulation of a given whisker equally affects the response to subsequent stimulation of the same, or a neighboring whisker 100 ms later (Figure 3F, *p* = 0.8 and 0.3 for the effect of PW and AW adaptation, respectively, Wilcoxon signed-rank test).

**Figure 4:**
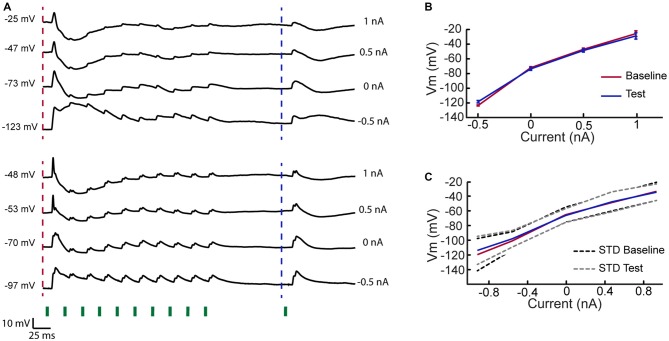
**Rapid adaptation of inhibition in VPM cells. (A)** Two example cells recorded at different holding currents while delivering 10 stimuli at 40 Hz to the PW followed by a test stimulation of the same whisker 100 ms later. Traces indicate that inhibitory input was evoked by first stimulus in adaptation train but rapidly adapted. At time of the test stimulation the membrane potential retuned to its resting value, measured prior to the first stimulus. **(B)** Mean membrane potential of upper example cell in **(A)** 10 ms before the first stimulation of the adaptation train and 10 ms prior to test stimulation (time points are indicated by dashed lines in **A**), measured for different holding currents. **(C)** As in **(B)** but averaged across five cells.

The process of adaptation in the VPM could involve local buildup of inhibition or intrinsic voltage dependent mechanisms. To test these possibilities, we averaged the membrane potential under different step currents in a subset of cells. Two example cells show that whisker stimulation evoked a substantial feedforward inhibition following the first stimulus (Figure 4A). However, inhibition rapidly adapted and the membrane potential returned to its baseline level at the time the test stimulation was delivered. This was valid for all step currents, indicating that inhibition was not accumulated during the adaptation train (see population mean in Figure 4C). These measurements also argue that intrinsic voltage dependent mechanisms are not involved in the adaptation process or in the response recovery.

### Adaptation of Multi-Whisker Upstream Cells in the PrV is Highly Specific

**Figure 5:**
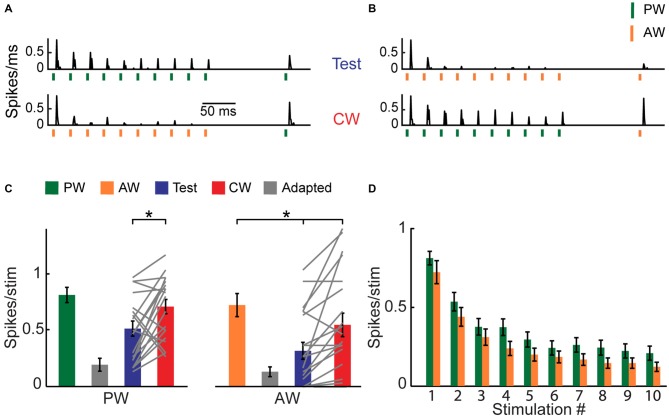
**CW adaptation of multi-whisker principal trigeminal nucleus (PrV) neurons. (A,B)** PSTHs (binned at 1 ms) of an example PrV neuron (*n* = 30 repetitions) to four different stimulation patterns. Stimuli are depicted below the traces by vertical bars. **(A)** The response to an isolated PW stimulation was examined 100 ms following a repetitive stimulation at 40 Hz delivered to the PW (test condition, *blue* shaded area; *upper trace*), or following repetitive stimulation of an AW (CW condition, *red* shaded area; *lower trace*). **(B)** Same as **(A)** but for the AW. **(C)** Population average (*n* = 20) of the number of spikes evoked per stimulus (20 ms bins) to control (*green and orange*), adapted (*gray*, 10th stimulus), test (*blue*) and CW (*red*) stimuli. Each gray line represents the values of a single unit. **(D)** Population average (*n* = 20) of the number of spikes evoked per stimulus (20 ms bins) in response to 10 stimulations of PW (*green*) and AW (*orange*) at 40 Hz. Asterisks (*) indicate significant differences (*p* < 0.05). Error bars represent SEMs.

Next, we investigated whether the non-specific adaptation that is observed in the VPM cascades from an earlier stage of tactile processing. Since the major sensory input to the VPM ascends from the PrV, we recorded single units in the PrV using metal electrodes and applied the same protocol as used for VPM cells with similar stimulation intensities. All recorded PrV cells exhibited a multi-whisker RF. Up to two clear units were separated from each site, and the whisker for which a given unit fired earlier on average was considered to be the PW (equivalent to the criteria that we used for VPM cells). The peri-stimulus time histograms (PSTHs) of an example PrV unit are presented in Figures 5A,B. Similar to VPM cells, this unit showed profound adaptation and a partial recovery of the test response. However, in contrast to the VPM, the CW response was less suppressed, indicating that adaptation was relatively specific to whisker identity. To quantify the test and CW responses across the population, for each unit we counted the total number of spikes that were evoked on average during the first 20 ms following a whisker deflection (Figures 5C,D). Similar to the example cell, we found that following adaptation the mean spike count to test stimulation was significantly smaller compared to the control response, whereas the spike count for CW stimulation was less affected by adaptation (Figure 5C; PW: control = 0.81 ± 0.05, test = 0.51 ± 0.1, CW = 0.71 ± 0.06, *n* = 20, *p* < 0.02, *left panel*; AW: control = 0.72 ± 0.10, test = 0.31 ± 0.15, CW = 0.54 ± 0.11, *n* = 20, *p* < 0.0005, *right panel*). Statistically, the effects were not completely symmetrical showing greater specificity for the AW (ASI = 0.16 ± 0.07 and 0.45 ± 0.09 for PW and AW respectively, *n* = 20, *p* = 0.011). In conclusion, the firing adaptation of PrV neurons is more specific compared to subthreshold responses of their downstream VPM cells, suggesting that the non-specific adaptation of VPM cells is not directly inherited from the PrV.

### Failure Analysis Suggests that Synaptic Depression Contributes Locally to Thalamic Adaptation

Our data demonstrate that PrV cells fire less than one spike per stimulus for most of the stimuli during adaptation train (Figure 5D). Considering the low number of fibers that innervate each VPM cell, some response failures are expected and their number should further increase during adaptation.

**Figure 6:**
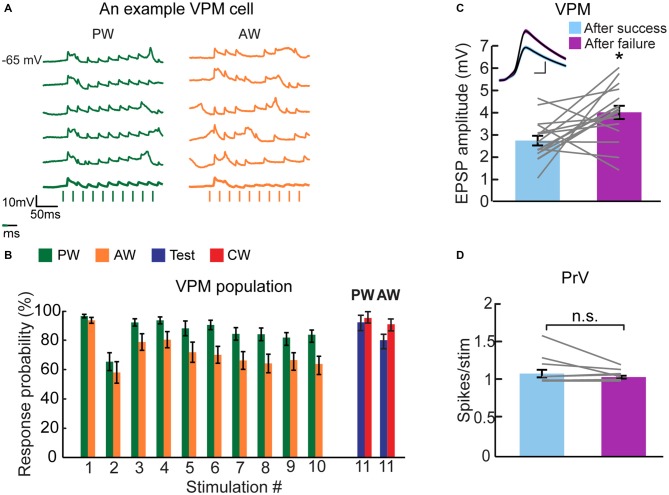
**Failure analysis suggests that synaptic depression contributes to thalamic adaptation. (A)** Individual traces of intracellularly recorded evoked responses of a multi-whisker VPM neuron to PW (*green*) and AW (*orange*) deflections (10 deflections at 40 Hz, depicted below by vertical bars). Average traces appear in bold lines. More failures are observed when the AW was stimulated (i.e., lower response probability) compared to PW stimulation. **(B)** Population average (*n* = 16 cells) of the response probability ratio plotted for multi-whisker VPM neurons to PW (*green*) and AW (*orange*) stimulation during adaptation train and in response to test (*blue*) and CW (*red*) stimulation. Values are normalized to first response. **(C)** Population average EPSP amplitude (*n* = 16 cells) of VPM neurons when preceded by an evoked EPSP (“after success”, in *pale blue*) or by an omitted response (“after failure”, in *purple*). Inset: example from a single neuron of overlaid average EPSPs (*black* lines) of all evoked responses after a success response and after a failure. Colored lines around the mean represent ± SEM. Scale bar of 1 mV/2 ms. **(D)** Population average (*n* = 14 units) of PrV neurons of spikes number evoked per stimulus (20 ms bins) in relation to the occurrence of spikes in the preceded stimulus; after a success response (*pale blue*) and after a failure (*purple*). Each gray line represents the values of a single comparison—some comparisons are obscured due to overlapping lines. Asterisks (*) indicate significant differences (*p* < 0.05). Error bars represent SEMs.

Indeed, whereas the first stimulus evoked highly reliable synaptic response in the VPM, complete synaptic failures were observed during repetitive whisker stimulation, in particular for the AW (example single trial traces of a VPM cell are depicted in Figure 6A). The population success rate (1-P_failure_) was over 95% for the first stimulus and it rapidly declined with adaptation, reaching ~85% and ~65% for the PW and AW, respectively (Figure 6B). However, the success probability for test and CW stimuli was kept relatively high (Figure 6B, blue and red bars).

Models of short-term synaptic depression (Tsodyks and Markram, 1997) predict that due to rapid recovery of synaptic resources, the response to a given stimulation should be enhanced if it followed a stimulation that failed to elicit any response (omitted response). To test this hypothesis, we averaged the successful synaptic responses of VPM cells to all stimuli based on whether a response was preceded by a success or an omitted response. An example cell (Figure 6C, inset) shows that the response to a given stimulation following an omitted response is 46% larger than the response that followed a successful response (following success = 3.37 ± 0.05 mV, *n* = 588; following omitted response = 4.92 ± 0.12 mV, *n* = 246, Mann-Whitney test, *p* < 0.0001). The similarity in the shape of the two averaged EPSPs indicates that other than a change in amplitude, the dynamics of the synaptic potential remained unchanged. In the population (Figure 6C), there was a significantly larger synaptic response when preceded by an omitted response (success = 2.78 ± 0.22 mV; failure = 4.08 ± 0.3 mV, *n* = 17 cells, Wilcoxon signed-rank test, *p* < 0.01). We repeated the analysis for PrV cells as this effect could arise from a higher firing probability in the upstream neurons. In the PrV, firing probability was independent of the occurrence or spike failure in the preceding stimulus (Figure 6D: success = 1.09 ± 0.04 spikes/stimulus, *n* = 14; failure = 1.05 ± 0.01 spikes/stimulus, *n* = 14, Wilcoxon signed-rank test, *p* = 0.92). These results strongly suggest that short-term synaptic depression of the trigemino-thalamic synapse is involved in the adaptation of VPM cells to repetitive whisker stimulation, a process that is likely to contribute to the low specificity of adaptation of VPM cells.

## Discussion

In this study, we used *in vivo* intracellular and extracellular recordings to reveal the transformation of stimulus-specific adaptation in the trigemino-thalamic synapse of the lemniscal pathway. We found that the firing and synaptic response of VPM cells to test stimulation of any whisker were equally suppressed by prior adapting stimulation of the same, or any other whisker within the RF of the cell. On the other hand, PrV cells exhibited a greater specificity, in particular for the AW. Therefore, we suggest that local thalamic mechanisms contribute to the generation of the non-specific adaptation. Finally, failure analysis strongly indicated that synaptic depression of brainstem inputs contributes to the adaptation pattern of thalamic cells. We suggest that during a sustained stimulation, multi-whisker thalamic cells scale down sensory inputs from different whiskers regardless of the exact tactile history that induces adaptation, expanding the notion of thalamic gating.

Suppressive interactions between thalamic responses were reported for paired stimulations at very short inter-stimulus intervals, up to about 50 ms (Higley and Contreras, 2007). In our study, the 100 ms interval from the last stimulus of the adaptation train to the test stimulation exceeds the time scale of recurrent inhibition (Lee et al., 1994) and of paired-pulse suppression (Fanselow and Nicolelis, 1999). Indeed, a paired stimulation of the same or a neighboring whisker at intervals of 100 ms or 350 ms, without prior adaptation, did not result in a significant suppression (Figure 3C). Hence, prior adaptation is required for the CW suppressive interactions.

Buildup of inhibition can strongly suppress the response to any whisker regardless the presynaptic organization. Similar to previous studies (Castro-Alamancos, 2002b), we find that inhibition rapidly adapts and that the membrane potential returns to resting levels prior to the test or CW stimulation (as evident in Figure 4). Hence, buildup of inhibition (Dealy and Tolhurst, 1974) is unlikely to contribute significantly to the strong reduction in the test or CW responses. Whisker-specific feedforward inhibition may also contribute to the suppression of the test and CW response, in particular if inhibition recovers from adaptation faster than excitation. Yet, similar to inhibitory inputs of cortical cells, where inhibition recovers slower than excitation (Cohen-Kashi Malina et al., 2013), we did not find a stronger inhibition to the test response compared to control stimulation (Figure 4). Intrinsic voltage-dependent conductances (Carandini and Ferster, 1997; Sanchez-Vives et al., 2000) are also unlikely to contribute to the CW suppression since these interactions were not affected by current injections that depolarized or hyperpolarized the cells by 10–20 mV. We cannot exclude, however, the possibility that presynaptic inhibition (Govindaiah et al., 2012; Lam and Sherman, 2013) or shunting inhibition of inputs at distal dendrites are involved.

Our results strongly indicate that short-term synaptic depression of the trigemino-thalamic synapse participates in VPM adaptation. Towards this end, we showed that the average synaptic response following an omitted response is larger compared to that evoked after a success (Figure 6C). While the increased EPSP following omitted response could in principal result from a higher response of brainstem neurons, a similar analysis indicates that PrV cells do not fire more action potentials after a complete spike failure (Figure 6D), supporting our conclusion that this depression occurs at the trigemino-thalamic synapse. Importantly, the failure rate in the VPM was substantially larger for the AW—as expected from the weaker response and stronger adaptation of PrV cells to AW stimulation (Figure 5D).

Synaptic depression of the trigemino-thalamic synapses of multi-whisker PrV cells may only partially explain the non-specific adaptation of VPM cells. Studies suggest that responses from different whiskers are conveyed by the same fibers (Deschênes et al., 2003). Accordingly, depression of the same trigemino-thalamic synapse is expected regardless of the whisker that was repetitively stimulated. However, since the response of PrV cells following PW stimulation is larger compared to AW stimulation (Figure 5), a greater adaptation is expected for PW stimulation, leading to a strong suppression of the response to subsequent stimulation of the AW. Hence, the effects of synaptic depression cannot be symmetrical (i.e., repetitive stimulation of the AW will entail less depression, resulting with smaller effect on the response to subsequent stimulation of the PW). Therefore, we argue that additional mechanisms are involved in generating the lower specificity of adaptation to whisker identity compared to the PrV, such as cortical feedback. Indeed, it was recently demonstrated that optogenetic activation of layer 6 of the barrel cortex alters the adaptation pattern of VPM cells (Mease et al., 2014).

Similar to the VPM, cells in layer 4 of the barrel cortex respond preferentially to stimulation of PW and to several AWs. The question of whether multi-whisker RFs arise from intracortical processing between neighboring cortical columns, or instead reflect convergence of information at earlier subcortical stages, is still being debated (Fox et al., 2003; Kwegyir-Afful et al., 2005; Katz et al., 2006; Hirata et al., 2009; Wright and Fox, 2010). Adaptation in cortical cells is highly whisker-specific (Katz et al., 2006), suggesting that they receive independent inputs from different whiskers. Given the global suppressive effect of adaptation in the thalamus, it is then puzzling how cortical adaptation can be whisker specific. In our study, 44% of thalamic cells recorded responded only to a single-whisker. Therefore, it is reasonable that adaptation in layer 4 is more specific compared to the thalamus.

The relay organization of brainstem inputs to the VPM shares several similarities with that of the retino-geniculate connectivity, in particular the small number of axons converging into a single thalamic cell (Cleland et al., 1971; Hamos et al., 1987; Mastronarde, 1992). This similarity suggests that thalamic adaptation may serve similar functions across modalities. Rats can move neighboring whiskers independently (Sachdev et al., 2002; Knutsen et al., 2006). This behavior together with the strong CW adaptation that we report in our study suggest that during natural whisking behavior, responses for a given whisker will depend on prior history of tactile stimulation caused by touch events made by other whiskers. Therefore, we suggest that during sustained stimulation, a global or non-specific form of thalamic adaptation indiscriminately decreases signals from multiple sources. While earlier studies showed that the thalamus gates sensory inputs to the cortex based on internal behavioral states (Halassa et al., 2014), our study expands this view by showing that individual thalamic cells dynamically gate their inputs by scaling down sensory inputs from different whiskers regardless of the exact whisker that was stimulated in recent history.

## Author Contributions

MJ and IL designed the study and wrote the article. MJ and BM performed the experiments (recordings of VPM cells were performed by MJ and recordings of PrV cells by BM). Data was analyzed by all authors.

## Conflict of Interest Statement

The authors declare that the research was conducted in the absence of any commercial or financial relationships that could be construed as a potential conflict of interest.
