# Supplementary figures and images for: The Transformation of Adaptation Specificity to Whisker Identity from Brainstem to Thalamus
Source: Front Syst Neurosci. 2016 Jun 23;10:56. doi: 10.3389/fnsys.2016.00056 (PMC4917531; doi:10.3389/fnsys.2016.00056)

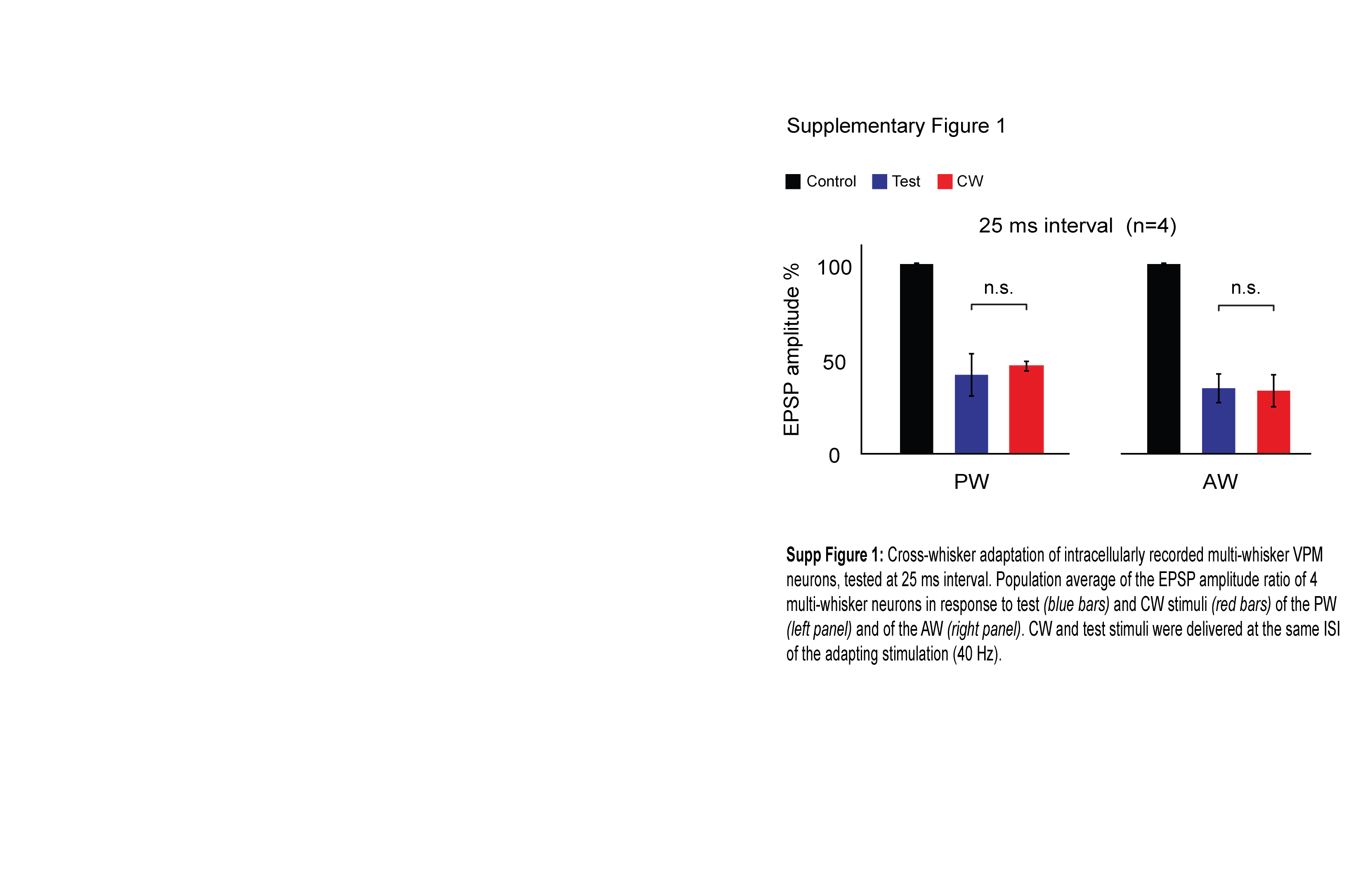

Supplement: Supplementary file 1 [file Image_1.tif]
